# Refractive index of human red blood cells between 290 nm and 1100 nm determined by optical extinction measurements

**DOI:** 10.1038/s41598-019-38767-5

**Published:** 2019-03-15

**Authors:** Jonas Gienger, Kathrin Smuda, Ralph Müller, Markus Bär, Jörg Neukammer

**Affiliations:** 1 Physikalisch-Technische Bundesanstalt (PTB), Abbestraße 2–12, 10587 Berlin, Germany; 2 Institute of Transfusion Medicine, Charité-Universitätsmedizin Berlin, Charitéplatz 1, 10117 Berlin, Germany

## Abstract

The knowledge of optical properties of biological cells is essential to interpret their interaction with light and to derive morphological information and parameters associated with cell function like the oxygen transport capacity of human red blood cells (RBCs). We present a method to determine the dependence between the refractive index (RI) of human RBCs and their intracellular hemoglobin (Hb) concentration from spectral extinction measurements of a cell suspension. The procedure is based on the analysis of the corresponding ensemble averaged extinction cross section ${\bar{{\boldsymbol{C}}}}_{{\bf{e}}{\bf{x}}{\bf{t}}}(\lambda )$. Thus far two complementary approaches have been taken to derive RIs of RBCs. The first one uses homogeneous macroscopic samples prepared by hemolysis for the destruction of the RBCs’ membranes and subsequent centrifugation. A second approach is the determination of RIs of single intact cells by microscopic investigation. These techniques are limited to a few discrete wavelengths or a rather narrow wavelength range. In addition most of these techniques require additional information about the concentration dependence. In contrast, our approach yields the RI increment with Hb concentration of intact, reversibly isovolumetrically sphered, oxygenated RBCs over a wide wavelength range from 290 nm to 1100 nm from macroscopic measurements.

## Introduction

The complex refractive index (RI) of biological cells describes their interaction with light and depends on the concentrations and spatial distribution of a variety of intracellular molecules, correlated to the corresponding biological function. For example, measurements of the cellular RI allow to derive the protein content in the cytoplasm of cells^1–3^. To support medical diagnosis and to elucidate fundamental cellular processes, various experimental methods are being applied. An overview on applications and experimental techniques in given in the review article by Liu *et al*.^4^. As summarized in this reference, the resulting information on cellular RI depends on the measurement technique and include the average RI of cells in suspension, the effective RI of a single cell and the two or three-dimensional RI mapping of a single cell^5^. Different diseases may cause local or integral changes of the cell RI, making RI a potential marker to differentiate between healthy and pathological cells. Such changes directly influence the light scattering properties of the cell and thus allow for detection of pathologies with optical means. This has been demonstrated, e. g., for precancerous epithelial cells^6,7^, pancreatic cells^8^, as well as red and white blood cells (RBCs and WBCs) from malaria patients^9–12^.

In particular, knowledge of the RI of RBCs is needed to derive their hemoglobin (Hb) content and corresponding cell volume from light scattering measurements in flow cytometry^13,14^. Such measurements are routinely performed for medical diagnostics in the complete blood count (CBC). Clinical parameters of the CBC include the mean corpuscular Hb concentration (MCHC), mean corpuscular volume (MCV) and the red cell distribution width (RDW) of a blood sample, that is, the coefficient of variation (relative standard deviation) of the volume of RBCs. These parameters allow, e. g., to discriminate between healthy and anaemic patients^15^. Among animal cells, mammalian RBCs have a particularly simple architecture, as they consist only of a liquid cytoplasm enclosed by a thin elastic membrane. Besides their important biological function – to transport oxygen in our blood – RBCs are an interesting model system for biophysics, since they do not possess a nucleus or organelles. RBCs are filled with a highly concentrated solution of the oxygen-transport metalloprotein Hb which dominates their optical properties as it amounts to about 98% of the RBCs’ solids^16^. Precise knowledge of the dependence between RI and intracellular Hb concentration is required for simulations^17–19^ and analysis^14,15,20,21^ of light scattering by single RBCs, to determine the intracellular Hb concentration from phase and holographic microscopy^3,22,23^, to understand the interaction of light with whole blood for purposes of clinical diagnostics^24,25^, or to visualise the appearance of tissues in computer graphics^26^.

Refractive index determination of RBCs and Hb solutions has been carried out already for many decades. Homogeneous solutions of Hb can be obtained from RBCs by breaking open the cell membranes (hemolysis) and it is known that their complex RI depends on the (intracellular) Hb concentration *c*_Hb_ according to^2,27,28^

1 $${\mathfrak{n}}(\lambda ,{c}_{{\rm{H}}{\rm{b}}})={{\mathfrak{n}}}_{{{\rm{H}}}_{2}{\rm{O}}}(\lambda )+{c}_{{\rm{H}}{\rm{b}}}[\alpha (\lambda )+{\rm{i}}\gamma (\lambda )],$$

where *α*(*λ*) is the increment of the real part of the RI or *real RI increment* and *γ*(*λ*) is the increment of the imaginary part of the RI or *imaginary RI increment*. The latter quantity is directly related to the molar attenuation coefficient, which is well known in the visible, near infrared (IR) and in the near ultraviolet (UV)^27,29^ as is the RI of water ${{\mathfrak{n}}}_{{{\rm{H}}}_{{\rm{2}}}{\rm{O}}}(\lambda )$^30,31^. On the other hand measurements of *α*(*λ*), even for a homogeneous bulk liquid are challenging with problems arising from the strong absorbance in this spectral range and from sample preparation at physiological concentrations exceeding *c*_Hb_ = 300 gL^−1^, due to high viscosity or incomplete dissolution of Hb^2^. For healthy persons typically the total Hb concentration in whole blood varies between 130 gL^−1^ and 160 gL^−1^ and the packed cell volume or hematocrit (HCT) ranges from 40% to 45%. Hence, the resulting intracellular Hb concentration lies between 300 gL^−1^ and 360 gL^−1^. These values correspond to molar concentrations of 19 mM and 22 mM relating to a single Hb subunit as commonly used in laboratory medicine or 4.7 mM to 5.6 mM relating to the tetrameric Hb molecule. Preparing homogeneous bulk liquids with such high concentrations by dissolving commercially available Hb powder is challenging, but concentrations up to 300 gL^−1^ have been reported in the literature^32,33^, while other researchers examining such samples preferred to use lower concentrations of 140 gL^−1 ^^34^. RI measurements were also presented for high-concentration bulk Hb solutions prepared from concentrated RBCs obtained from fresh blood samples which were hemolysed by freezing. For these samples, Hb concentrations were in the 260 gL^−1^ to 306 gL^−1^ range^27,28,35^. Due to the aforementioned challenges and possibly also due to different types of samples used (i. e., obtained from either dissolved Hb powder or fresh RBCs), the values reported for *α*(*λ*) by various researchers differ by more than 30%. In the 1950 s Barer and Joseph^1,2^ compiled and reported values of *α* ≈ 0.19 mLg^−1^ for Hb solutions in the visible range, without resolving the wavelength dependence. These values have been widely used in simulation and analysis of light scattering and microscopic data^14,22,36,37^. Some more recent experiments confirmed these values in the visible an near IR^35^, whereas deviating results were obtained by other authors. Two recent studies^33,34^ found values of *α* ≈ 0.15 mLg^−1^ in the visible range. In contrast, significantly higher values of *α* ≈ 0.26 mLg^−1^ were reported by Friebel and Meinke^27,28^ in a wider spectral range from 250 nm to 1100 nm. These values have been widely used in application-oriented investigations^3,10,15,24,38^. Recent studies employing microscopic techniques on Hb solutions in the visible^32^ and single RBCs in the UV^39^ reported values of *α* ≈ 0.23 mLg^−1^.

For practical applications, such as light scattering techniques and microscopy, the optical properties of interest are those of intact RBCs and usually not those of artificially produced Hb solutions. The two need not necessarily coincide quantitatively, even if similar features have been found for their wavelength dependence^39,40^. Equation (1) is believed to provide a suitable model for the RI intact RBCs because the membrane is very thin compared to the wavelength of visible light^14^ and has a low volume fraction^16^. In addition to the intrinsic complexity of measuring the optical properties of single microscopic cells compared to bulk liquids, the task is further complicated by the fact that the intracellular concentration *c*_Hb_ is a priori unknown, since it varies by about 6–8%^14,41^ between the cells of a healthy individual. In studies where the RI of single intact RBCs was measured, data analysis either required a priori knowledge about *α*(*λ*)^3,22,23,32,41^ or the concentration was eliminated by considering relative RI changes^40^. A very recent study presented spectral microscopic measurements of the complex RI of single cells^39^, thus enabling the determination of *α* using the known *γ*.

In this paper we present an approach to determine the real RI increment *α*(*λ*) from intact cells. Employing extinction spectroscopy, we use a relatively simple ensemble measurement technique. In contrast to measuring single cells, this eliminates the problem of a priori knowledge about the intracellular Hb concentration *c*_Hb_, since for a blood sample, the mean Hb concentration (MCHC) averaged over all cells can be easily measured. Compared to measurements of highly concentrated Hb solutions, sample handling is much easier for dilute RBC suspensions. The method we developed to determine the complex RI of biological cells in cell suspensions is applied to determine RIs of sphered RBCs between 290 nm and 1100 nm. To this end we modified a procedure recently proposed and tested for non-absorbing polystyrene microspheres^42^. The method is based on measurement of collimated transmittance of a dilute cell suspension. This allows to extract the ensemble-averaged extinction cross section of the suspension in dependence on wavelength. We use sphered RBCs, hence the measured extinction spectra can be analysed using the analytical solution for the scattering of light by a sphere (Mie solution^43,44^) and *α*(*λ*) can be extracted along with the distribution of cell size and *c*_Hb_ by nonlinear least-squares optimisation of a suitable parameter set.

## Results

Human RBCs were isolated from fresh blood samples by washing as described in the “Materials and methods” section. RBCs in suspension were sphered using a chemical treatment that reduces the membranes’ area while leaving them intact and not affecting the inside of the cell^13,14^. The sphering occurs only as long as the cells are suspended in a specific reagent and is reversed when it is replaced by isotonic saline. Measurements of the collimated transmittance *T*(*λ*) of dilute RBC suspensions were performed in dependence on the vacuum wavelength *λ*. From these we computed the ensemble-averaged extinction cross section ${\bar{C}}_{{\rm{e}}{\rm{x}}{\rm{t}}}(\lambda )$ according to

2 $${\overline{C}}_{{\rm{ext}}}(\lambda )=-\,\mathrm{ln}\,[T(\lambda )]\,\frac{1}{d\,c},$$

where *c* = *c*_RBC_/*ϕ* is the concentration of RBCs in the sample (diluted by a factor *ϕ*) and *d* = 10 mm is the thickness of the cuvette. The extinction cross section *C*_ext_(*λ*) of a single particle or cell describes how much light is removed from a beam of light by scattering and absorption processes. The measured quantity ${\bar{C}}_{{\rm{e}}{\rm{x}}{\rm{t}}}(\lambda )$ is the ensemble average over all cell sizes and intracellular Hb concentrations in the sample.

**Figure 1:**
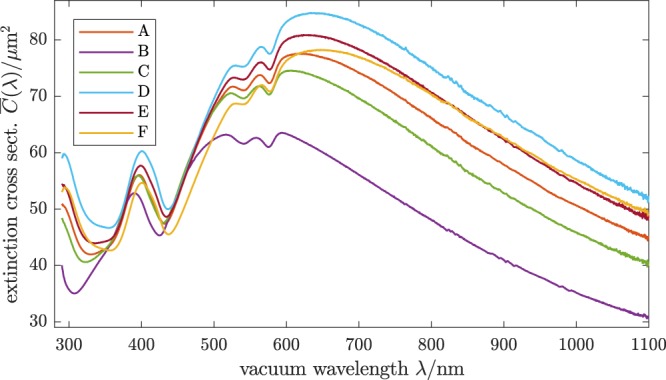
Extinction cross sections of sphered RBCs, washed three times and suspended in sphering reagent. Samples from six volunteers A–F who exhibit significantly different MCV were investigated. The curves shown are rescaled according to the optimisation results to eliminate the effect of particle concentration errors [rescaled by 1/(1 + *η*), cf. Eq. (8)]. Underlying data are available as Supplementary Data S1.

Measured spectral extinction cross sections ${\overline{C}}_{{\rm{ext}}}(\lambda )$ of sphered RBCs from six volunteers (A, B, C, D, E, F), recorded at room temperature are shown in Fig. 1. The six samples exhibit significant variation of the extinction cross sections while some features reveal similarities. Since extinction is the combined effect of scattering and absorption of light, the observed spectral features of the extinction cross sections can be interpreted as a combination of resonances due to Mie scattering (caused mainly by the size of the cells and the real part of their RI) and the absorbance bands of Hb (related to the imaginary part of their RI). The highest values for the spectral extinction cross section are observed for sample D with the largest RBCs (MCV = 99.5 fL). The cross section generally decreases for the samples with RBCs of slightly smaller volume and is significantly lower for sample B, which has by far the smallest volume RBCs (MCV = 62.3 fL). The absorption of Hb within the cells is clearly discernible as a single peak around 420 nm (Soret band) and a double peak at 550 nm and 580 nm (Q bands). It is conspicuous that the absorption results in an increased extinction cross section for the Soret band while the cross section is reduced by the absorption of the Q bands. This observation is explained by the fact that the Mie resonances are caused by interference, which are damped by absorption, i. e., a non-zero imaginary part of the particle RI. For a single non-absorbing particle with size somewhat larger than the wavelength, the extinction cross section as a function of wavelength *C*_ext_(*λ*) oscillates around the value of 2*πR*^2^, where *R* is the particle radius, i. e., two times the geometrical cross section. A non-zero imaginary part of the particle RI dampens these resonances towards the geometrical limit, i. e., shifts *C*_ext_(*λ*) towards 2*πR*^2^ at absorption bands (e. g., 2*πR*^2^ ≈ 47 μm^2^ for an average RBC of volunteer A). Because for the RBCs examined here the Soret band is located near a minimum of the Mie resonances, *C*_ext_(*λ*) is increased compared to non-absorbing particles. At the Q bands on the other hand, which are located near a maximum, *C*_ext_(*λ*) is decreased. However, one should note that also the dispersion of the real part of the RI has a contributions to the shape of *C*_ext_(*λ*). It is also evident from Fig. 1 that the observed maxima or minima in *C*_ext_(*λ*) are slightly shifted with respect to absorption spectra of (non-scattering) oxyhemoglobin solutions. This effect is caused by the combination of scattering and absorption and not due to contributions of other Hb variants. Our interpretation that only negligibly small perturbations occur due to the presence of other Hb variants is supported by the data analysis, since all extinction spectra can be described using the same absorption spectra of oxyhemoglobin^27^ together with the hematological parameters from the CBCs of the blood samples listed in Table 1.

**Table 1:** Properties of concentration distribution and size distribution of RBC samples obtained from the CBC of whole blood and used as additional information in the optimisation (left) in comparison to the optimisation results (right). MCHC = mean(*c*_Hb_), HDW = std(*c*_Hb_)/mean(*c*_Hb_), ${\rm{MCV}}={\rm{mean}}(V)=\frac{4\pi }{3}\,{\rm{mean}}({R}^{3})$, RDW = std(*V*)/mean(*V*), where std denotes the standard deviation. *c*_RBC_ is the RBC concentration given for the fresh whole blood sample and the washed RBCs and *ϕ* the dilution factor applied for the extinction measurement of the washed RBCs. The numbers in parentheses are the estimated standard uncertainties, referred to the last digit.

Volunteer	CBC					*ϕ*	Optimisation Result		
	*c*_RBC_/pL^−1^		$\frac{{\bf{MCHC}}}{{\bf{g}}{{\bf{L}}}^{-{\bf{1}}}}$	$\frac{{\bf{MCV}}}{{\bf{fL}}}$	$\frac{{\bf{RDW}}}{{\boldsymbol{ \% }}}$		$\frac{{\bf{HDW}}}{{\boldsymbol{ \% }}}$	$\frac{{\bf{MCV}}}{{\bf{fL}}}$	$\frac{{\bf{RDW}}}{{\boldsymbol{ \% }}}$
	fresh	washed							
A	4.62	4.58	329 (6)	86.0 (1.0)	12.7 (1.0)	1670	3.7(4)	85.7(1.0)	12.7 (1.0)
B	6.55	4.02	324 (10)	63.0 (2.2)	15.7 (1.2)	1430	6.4(3)	66.0(2.2)	15.8(1.2)
C	4.42	4.47	331 (6)	81.5 (1.0)	15.2 (1.0)	3240	4.6(5)	81.5(1.0)	15.2(1.0)
D	4.04	4.48	352 (6)	99.5 (1.0)	12.5 (1.0)	3240	4.6(3)	98.2(0.9)	12.5(1.0)
E	4.28	5.19	336 (6)	91.3 (1.0)	12.3 (1.0)	3240	3.5(5)	91.1(1.0)	12.3(1.0)
F	4.80	4.05	352 (6)	89.5 (1.0)	12.1 (1.0)	1620	4.2(3)	86.5(1.0)	12.0(1.0)

The optical properties of the RBCs were extracted using a nonlinear least-squares approach. In a nutshell, the data analysis requires minimising the cost function

3 $${\chi }^{2}({\boldsymbol{\psi }})\,:\,=\sum _{i=1}^{N}{w}_{i}\,{[ {\mathcal M} ({\lambda }_{i};{\boldsymbol{\psi }})-{y}_{i}]}^{2}+\sum _{j=1}^{2}{w}_{j}^{{\rm{CBC}}}{[{ {\mathcal M} }_{j}^{{\rm{CBC}}}({\boldsymbol{\psi }})-{z}_{j}]}^{2}\mathrm{.}$$

Here ${\mathcal M} ({\lambda }_{i};{\boldsymbol{\psi }})$ is a mathematical model of the extinction cross section for wavelengths *λ*_1_, …, *λ*_*N*_ and *y*_*i*_ are the corresponding measurement data (Fig. 1). The parameter vector ***ψ*** contains coefficients characterizing the real RI increment *α*(*λ*) as well as parameters characterizing the distributions of RBC sizes and intracellular Hb concentrations. The secondary model ${ {\mathcal M} }^{{\rm{CBC}}}({\boldsymbol{\psi }})$ computes the size distribution parameters (MCV, RDW) and *z*_*j*_ are the corresponding independent measurements of these values (Table 1). Weights *w*_*i*_ and ${w}_{j}^{{\rm{CBC}}}$ account for measurement uncertainties. The second term in the above equation implements the use of the RBCs’ mean volume (MCV), along with the volume distribution width (RDW) as additional information in the analysis of extinction spectra, because we found that without this additional information unambiguous parameter retrieval is not possible. For the same reason, the mean of *c*_Hb_ (MCHC) was held constant at the value from the CBC during optimisation. This is not because the model is insensitive to these parameters individually, but because they have similar effects on the measured spectrum, e. g., a too high MCHC or MCV may compensate for a too low *α*(*λ*). In turn, the values of these hematological parameters can be computed from the optimised parameter vector ***ψ***. Details of the data analysis are described in the section “Materials and methods”. The results of the optimisation are shown in Table 1. But because of the described effect of mutually compensating parameters in the model, the MCV and RDW mean values and uncertainties found by optimisation basically reflect the input CBC parameters and their respective uncertainties. In addition, the width of the intracellular Hb concentration distribution HDW = std(*c*_Hb_)/mean(*c*_Hb_), which is not part of the standard CBC was also retrieved.

**Figure 2:**
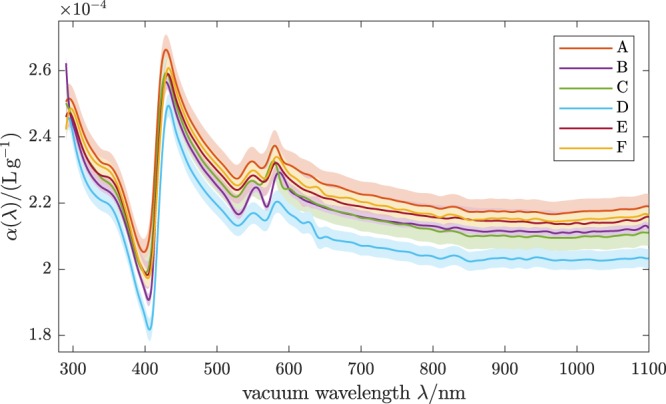
Real RI increment of oxygenated human RBCs obtained the extinction cross sections in Fig. 1. Shaded bands indicate ±1 estimated uncertainties, accounting for noise in the analysed spectra and uncertainties of CBC parameters. Underlying data are available as Supplementary Data S1.

**Figure 3:**
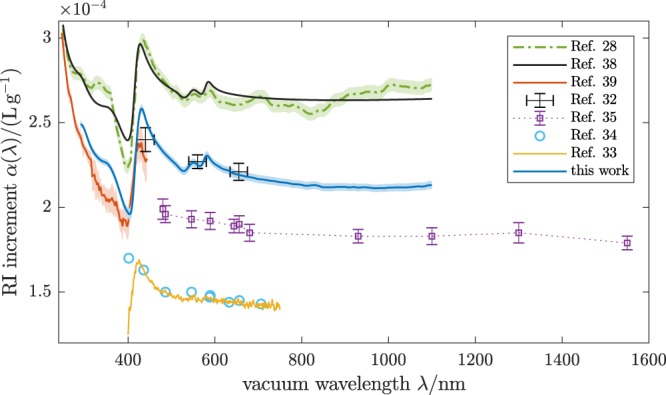
Real RI increment of oxygenated human RBCs. The blue line is the arithmetic mean of the individual curves in Fig. 2 (underlying data available as Supplementary Data S1). Various literature values for the RI increment of oxygenated Hb solutions and RBCs are shown for comparison. Samples for these measurements were: (i) Hb solutions from powder^32–34^, (ii) Hb solutions from freshly hemolysed RBCs^28,35^ and (iii) single native RBCs^39^. The Kramers-Kronig computation^38^ matched to Ref.^28^ shows the RI dispersion expected from the accurately known extinction spectrum *γ*(*λ*).

The real RI increment *α*(*λ*) obtained by nonlinear optimisation is shown in Fig. 2. Even though the RBC samples examined show huge individual variability and hence the underlying ${\overline{C}}_{{\rm{ext}}}(\lambda )$ differ significantly, the *α*(*λ*) have the same wavelength dependence within their respective estimated uncertainties, except for a small offset. All six curves lie around *α* ≈ 0.22 mLg^−1^, with differences of about 0.015 mLg^−1^ being present between the highest curve (A) and the lowest (D) for most wavelengths, however reaching 0.036 mLg^−1^ at the Soret band around 420 nm. This agreement is remarkably good, given that the estimated standard uncertainties (shaded bands in Fig. 2) account only for effects of noise in the measured extinction spectra and uncertainties of the parameters from the CBC. To assess the influence of a partial deoxygenation, we repeated the data analysis assuming the absorption spectrum of a mixture of 10% deoxygenated Hb (deoxyHb) and 90% oxygenated Hb (oxyHb)^27^ for the imaginary RI increment *γ*(*λ*) of the RBCs. The difference in the resulting *α*(*λ*) is highest near the Soret band around 420 nm and the Q bands between 550 and 600 nm and remains below 0.01 mLg^−1^ for all wavelengths. Moreover, for a given dataset the optimisation assuming 10% deoxyHb does not result in a systematic shift of the *α*(*λ*) curve compared to the analysis assuming full oxygenation. It does, however, generally lead to a poorer fit. Hence, we conclude that the shifts between the curves in Fig. 2 are not caused by different levels of Hb variants in the RBCs and that oxygenation levels in all samples were significantly higher than 90%. The observed systematic deviations might be caused by small systematic errors in the optical setup, which are not included in the error model, possible scattering effects of residual WBCs or blood platelets in the washed RBC samples or by different intra-RBC concentrations of proteins other than Hb. In addition, model errors due to the class of functions assumed for the distributions of size and Hb distribution are a possible cause for the deviations. To account for these influences, too, as a final result for the real RI increment of oxygenated RBCs the average of the six curves was taken and the uncertainty estimated from the sample variance, which is shown as the blue curve in Fig. 3.

## Discussion

Our measurements demonstrate a significant variation of the spectral extinction cross sections observed for six individuals. Despite these large differences, our analyses reveal that within the uncertainties of the experiment and the model the same dependence of the spectral RI on intracellular Hb is derived, described by the increment *α*(*λ*) ≈ 0.22 mLg^−1^ of the real part of the RI. Compared to the widely used values reported by Barer and Joseph^1,2^ of *α* ≈ 0.19 mLg^−1^ for Hb in the visible range our results for the real RI increment of oxygenated RBCs (blue curve in Fig. 3) of *α*(*λ*) ≈ 0.22 mLg^−1^ are significantly higher. On the other hand, our results are in good agreement with values measured from a single native RBC using hyperspectral microscopy^39^ for wavelengths *λ* ∈ [250, 440] nm (red curve in Fig. 3) while exhibiting a less noisy profile with lower uncertainties and covering a wider wavelength range *λ* ∈ [290, 1100] nm. Good agreement is also found for values measured for solutions prepared from Hb powder using spectroscopic phase microscopy^32^ at discrete wavelengths *λ* ∈ [440 nm, 700 nm] (black crosses in Fig. 3). As a side note the authors of Ref.^32^ reported a lower RI increment of *α*_BSA_ = 0.18 mLg^−1^ for bovine serum albumin (BSA) which, in contrast to the value for Hb, is consistent with those values of Barer and Joseph^1,2^.

The values we obtained for *α*(*λ*) are about 0.05 mLg^−1^ or 20% lower than values measured by Friebel and Meinke for homogenized RBC cytoplasm, obtained by hemolysis^28^ (green dash-dotted curve in Fig. 3). At the same time the peak-peak amplitude Δ_pp_*α* = 0.060 mLg^−1^ of the dispersion feature around *λ* ≈ 420 nm is lower by the same percentage. Comparing to the measurements of Lazareva and Tuchin^35^ (purple dotted line in Fig. 3) for samples prepared from fresh RBCs in a similar way as those in Ref.^28^ our results for *α*(*λ*) are about 0.03 mLg^−1^ higher. These deviations in both directions are explainable by a scaling error of some of the curves, most likely stemming from the measurement of the Hb concentration. Another possible source for these discrepancies is the formation of Hb-enriched or depleted layers near interfaces, as these measurements were performed at solution-air interfaces^28^ and solution-glass interfaces^35^, respectively.

The overall wavelength-dependence of our result for *α*(*λ*) closely resembles the curves computed using the Kramers-Kronig (KK) transformation of the absorption spectrum of Hb solutions^38,45^ (black curve in Fig. 3). In particular, the peak-peak amplitude around *λ* ≈ 420 nm differs by less than 5%. However, the absolute value of *α*(*λ*) is lower for our measurements than for the KK results, since for the latter the unknown constant background was fitted to data from Ref.^28^. If the KK analysis of Ref.^38^ is repeated with the background fitted to the results of the present work, very good agreement within the estimated uncertainties is found between the two curves. This observation indicates that the RI of RBCs is indeed practically identical to that of an aqueous Hb solution of equal concentration. It also justifies the use of imaginary RI increment data obtained from absorption spectra of Hb solutions in our analysis of RBC extinction spectra.

As presented in Ref.^38^, a KK analysis also allows to compute the real RI increment for other Hb variants such as deoxyHb. From the KK analysis, a significant difference of the wavelength dependence of the real RI increment *α*(*λ*) is expected between oxyHb and deoxHb, especially around the Soret and Q bands. A mixture of both variants will have an intermediate RI increment. On the other hand, the KK analysis predicts that the wavlength-averaged absolute value of the real RI increment in the near UV to near IR hardly differs between the Hb variants, because it is linked to the strong deep UV absorbance of the peptide bonds in the protein chains, which are chemically identical between the Hb variants. In the present study, we assumed that the vast majority of the Hb in the RBCs is oxygenated. This assumption is confirmed by a comparison of absorption spectra of hemolysed RBCs with literature values for oxygenated Hb solutions, indicating oxygen saturation of at least 90%. Furthermore, levels of other Hb variants were assessed with a blood gas analyser (see “Materials and methods” section). If relevant levels of deoxyHb or other Hb variants were present, we would wrongly attribute the result for *α*(*λ*) to fully oxygenated RBCs. More importantly, the absorption spectrum of (oxygenated) Hb is part of the mathematical forward model and thus has an influence on data analysis itself. We assessed the latter by repeating the data analysis assuming 10% deoxyHb, which yielded a poorer fit.

We employed a hematology analyser to determine the mean intracellular Hb concentration (MCHC). These devices are used in laboratory medicine and undergo frequent external controls and calibration in order to yield reliable values for medical diagnoses. Furthermore the RBCs remained intact over the course of the experiment. This means that errors in the number concentration of the cells can occur during preparation, but are easily compensated for during data analysis. On the other hand changes of the MCHC are unlikely, as this would require loss of cells of only a certain intracellular Hb concentration. Thus an error of the MCHC of more than 2% is not expected for our findings, which was accounted for as uncertainty of the additional information from CBC in the parameter optimisation.

Comparing to other results for the RI increment of Hb solutions, our findings are incompatible with the data presented in Refs.^33,34^ (cyan circles and yellow line in Fig. 3), where values of *α*(*λ*) ≈ 0.15 mLg^−1^ for *λ* ∈ [400 nm, 750 nm] were reported for solutions prepared from human and bovine Hb in dry form and concentrations determined from the weighed-in protein mass. The limitations of this method over spectroscopic concentration determination have been discussed^2^. Furthermore, the solutions created from dry Hb, being in the form of non-functional methemoglobin (metHb), require conversion to oxyHb using sodium bicarbonate. This may, at least in part, explain the discrepancies.

The measurement technique along with the data analysis method presented here provide a more precise approach for future determination of the optical properties of approximately spherical biological entities such as RBCs in different oxygenation states or chemical environments (e. g., incubated with glucose^46^), Hb based blood subsitutes^47^, other animal cells^48^, or phytoplankton^49^. Besides Mie scattering by homogeneous spheres, efficient numerical light scattering simulation tools exist for concentric spheres^44^ (e. g., a model for lymphocytes) or spheroids^50^ (e. g., a simplified model for rod-shaped E. coli bacteria, blood platelets or native RBCs). For more general shapes, T-matrix methods^51^ can be used to compute particle extinction cross sections. Hence, our data analysis method is applicable to such objects, too. In particular, the spectral RIs of other blood cells could be determined from ensemble measurements of suspensions prepared by established enrichment and purification techniques, i. e., magnetic or flow cytometric cell sorting. This will enable investigations of differences between normal and pathologically modified cells like infested RBCs in malaria patients.

## Materials and methods

### Sample preparation

Freshly withdrawn venous blood, anti-coagulated by EDTA (1.8 gL^−1^), was collected from six healthy, non-smoking volunteers (A, B, C, D, E and F) with the vacutainer system from BD (BD, Heidelberg, Germany) and immediately processed. Informed consent was obtained from all donors in written form. The blood samples were withdrawn in accordance with the transfusion law of Germany. The use of donor blood samples for scientific purposes was approved by the ethics committee of the Charité–Universitätsmedizin Berlin (# EA1/137/14). The blood samples were characterised by determining the complete blood counts (CBCs) using a XS 800i hematology analyser (SysmexEurope GmbH), located in the central laboratory. In healthy individuals, free Hb concentrations in blood serum are less than 0.1%^52^ and negligible for the analysis of our measurements. A blood gas analyser (ABL 725, Radiometer GmbH, Germany) served to determine the relative metHb and carbomonoxy-Hb (COHb) concentrations, being <1.2% and <2.5%, respectively, for the six samples investigated. Subsequently, for WBC and platelet depletion, 10 mL of whole blood were washed three times (150 g, 5 min) in 50 mL of phosphate buffered saline (PBS; sigma, Germany). Washed RBCs were re-suspended in PBS to a final volume of 10 mL. To control the washing cycles the concentrations of RBCs, WBCs and platelets were measured on site before and after each washing step by an ABX Micros 60 analyser (Axon Lab AG, Germany). In this way we ensured that WBC and platelet concentrations are low and extinction spectra of RBCs are not distorted. The sphering reagent is a commercially available substance (CELL-DYN diluents/sheath reagent, Abbott GmbH & Co. KG, Diagnostik, Germany) used for the isovolumetric sphering of RBCs in hematology analysers. For sphering, washed RBCs were diluted 1:100 in the sphering reagent. This pre-diluted suspension was used as stock solution for dilution series.

Typically, the preparation took 90 minutes. Since during this time the sample was exposed to atmospheric oxygen (i. e., *p*O_2_ = 212 hPa = 159 mmHg), saturation is reached and the deoxygenated Hb variant is converted to oxygenated Hb. Time constants for the oxygen uptake amount to about 500 ms for 50% saturation^53^. In Ref.^53^ it was also shown the this time reduces with decreasing HCT value, i. e., the reaction is accelerated when RBCs are diluted as in our measurements. Absorption spectra of lysed RBCs were measured with the same experimental setup used for extinction measurements. They were found to agree with literature data for oxyHb^27,29^ up to the concentration error from volumetric dilution. Hence we can use the literature values of oxygenated Hb^27^ for *γ*(*λ*) in our analysis.

### Experimental setup

**Figure 4:**
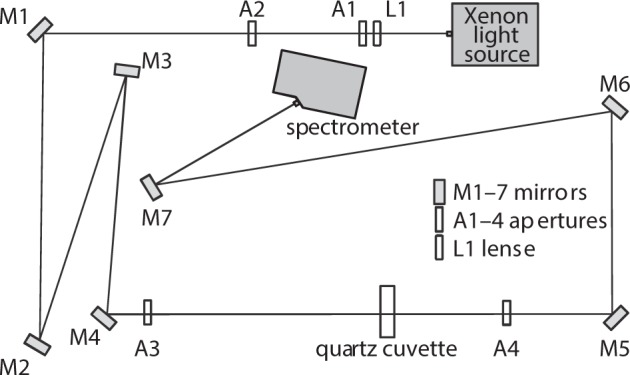
Optical layout to measure extinction spectra.

The optical setup for spectral transmittance measurements is shown in Fig. 4. A high-power, continuous HPX-2000 Xenon light source is applied to irradiate the sample in the wavelength range between 185 nm to 2000 nm. For spectral analysis between 200 nm and 1100 nm, a Maya2000 Pro spectrometer was used (Ocean Optics, Inc., USA). With the help of 4 mirrors M1–M4 the folded light path features a length of approximately 2.5 m between the light source and the sample cuvette. The lense L1 is used as condenser to obtain an approximately parallel light beam. The apertures A1–A3 serve to reduce the size of the beam to a diameter of about 1 mm corresponding to a divergence of 0.2 mrad or 0.01° (half angle). The samples are filled in a quartz cuvette (Hellma Analytics, Germany) with *d* = (10 ± 0.01) mm optical path length. Aperture A4 blocks the light scattered in the non-forward direction by the sample to suppress background light. The spectrometer is placed 1.5 m from the sample via mirrors M5–M7 and equipped with an entrance slit of 50 μm width and 1.0 mm height. The slit width results in a spectral resolution of approximately 0.45 nm, the slit height corresponds to an observation angle of 0.3 mrad or 0.02 (half angle). The long distance of 1.5 m between the cuvette and the detector serves to effectively suppress light scattered at small angles into the spectrometer’s aperture. This allows to neglect unwanted contributions to the directed transmittance when analysing the measurements. The experimental setup in Fig. 4 allows to measure the same quantity as the monochromator-based setup previously used by Gienger *et al*.^42^ to validate the method for spectral RI determination of particles. However, the setup in Fig. 4 offers a significant advantage, since due to the parallel detection of the spectrum in contrast to the previously used wavelength scanning, the measurement time is reduced from typically 20 min to about 10 s.

### Measurement of transmittance and calculation of extinction cross sections

For transmission measurements, pre-diluted sphered RBCs were further diluted with the sphering reagent and the spectral intensity ${I}_{{\rm{sample}},j}(\lambda )$ was measured for 6 to 8 different dilutions per sample. The number concentrations *c*_*j*_ of the cells were selected such that the transmittance ${T}_{j}(\lambda )={I}_{{\rm{sample}},j}(\lambda )/{I}_{0}(\lambda )$ ranged from roughly 95% down to 30%. *I*_0_(*λ*) is the null measurement where the cuvette filled with the sphering reagent only. The offset due to dark counts and read out procedure of the diode array were subtracted from all spectra. This measurement of concentration series enables us to exclude multiple scattering effects and to compute the extinction cross section ${\overline{C}}_{{\rm{ext}},j}(\lambda )$ according to

4 $${\overline{C}}_{{\rm{ext}},j}(\lambda )=-\,\mathrm{ln}\,[{T}_{j}(\lambda )]\,\frac{1}{d\,{c}_{j}},$$

as this formula implies extinction caused by single scattering. Since the ${\bar{C}}_{{\rm{e}}{\rm{x}}{\rm{t}},j}(\lambda )$ curves thus computed lie on top of each other inside the measurement accuracy, multiple scattering can be excluded.

The volumetric dilution by adjustable pipettes contributes to the uncertainty of the concentrations *c*_*j*_ of an estimated 2–4%, depending on dilution. Accounting for the accuracy of hematology analysers, the RBC concentrations of the undiluted samples have a relative uncertainty of about 4%. It follows that ${\bar{C}}_{{\rm{e}}{\rm{x}}{\rm{t}},j}(\lambda )$ is only measured up to a prefactor corresponding to the relative error of the number concentration of cells in the diluted sample, which accumulates to approximately 6%. However, even larger concentration errors are easily accounted for in the data analysis as described in section “Mathematical model” (see Eq. (8)). The concentration series were recorded such that the cuvette was not moved between measurements: Increasing volumes of the RBC suspension were added to the fluid-filled 10 mm cuvette (starting with 2.2 mL of sphering reagent) and mixed by pipetting back and forth and using the magnetic stir bar for homogenisation. Care was taken not to touch the cuvette walls in the process, as not to change the angle relative to the incident beam. This minimises errors from light reflected at the cuvette and avoids artefacts due to displacement of the transmitted light when tilting the cuvette.

### Optical properties of materials

Using an Abbe refractometer (ORT 1RS, Kern Optics, Germany) the real part of the RI of the sphering reagent was measured at *λ* = 590 nm and found to be higher than that of pure water by Δ*n* = 0.0020(3). Furthermore, the absorption spectrum was recorded with the setup in Fig. 4. An absorption band was found between 220 nm and 290 nm with a peak in the imaginary part if the RI of 1 × 10^−5^. This limits the lowest wavelength in our analysis to 290 nm since the transmittance in a 10 mm cuvette drops down to about 1.2% compared to water at the absorption peak, which results in a very low signal to noise ratio.

### Mathematical model

The measured spectral extinction cross sections ${\overline{C}}_{{\rm{ext}}}(\lambda )$ depend on the quantity to be determined, *α*(*λ*), in a complicated nonlinear way. Hence, data analysis requires solving an inverse problem: Find those optical properties of RBCs (and their size and concentration distribution) that explain the data. To solve this problem by nonlinear numerical optimisation a *forward model* is needed to compute ${\overline{C}}_{{\rm{ext}}}(\lambda )$ for a given parameter set.

Firstly, we define the ensemble average

5 $${\overline{C}}_{{\rm{ext}}}(\lambda )={\int }_{0}^{\infty }{\int }_{0}^{\infty }C(\lambda ;{c}_{{\rm{Hb}}},R)\,q({c}_{{\rm{Hb}}})\,r(R)\,{\rm{d}}{c}_{{\rm{Hb}}}\,{\rm{d}}R,$$

where *C*(*λ*; *c*_Hb_, *R*) is the extinction cross section of a single cell of radius *R* and intracellular Hb concentration *c*_Hb_. The cell’s refractive index is given by Eq. (1) and the RI of the surrounding medium (sphering reagent) is ${n}_{{\rm{m}}}(\lambda )\in {\mathbb{R}}$. The size distribution in the blood sample is given by *r*(*R*) and the distribution of the intracellular Hb concentration *c*_Hb_ is given by *q*(*c*_Hb_). Measurements on single RBCs suggest that *R* and *c*_Hb_ are statistically independent^14,41^, thus motivating a separation of *q* and *r* in our treatment.

The Mie solution allows for efficient computation of *C*(*λ*; *c*_Hb_, *R*). The known quantities in Eq. (1) are ${{\mathfrak{n}}}_{{{\rm{H}}}_{{\rm{2}}}{\rm{O}}}$ and *γ* for which we use literature values^27,31^. Furthermore we assume ${n}_{{\rm{m}}}(\lambda )={n}_{{{\rm{H}}}_{{\rm{2}}}{\rm{O}}}(\lambda )+0.002$ for all *λ*, as this RI difference was measured for the sphering reagent at 590 nm. As presented in Ref.^42^, we expand the unknown function *α*, describing the wavelength-dependent increment of the real RI with concentration, into a finite series

6 $$\alpha (\lambda )=\sum _{j=1}^{M}{a}_{j}\,{g}_{j}(\lambda )$$

with real coefficients *a*_*j*_, where the *g*_*j*_ are orthonormal basis functions. Here, we use a set of orthonormalized third-order cardinal splines with a uniform grid spacing of Δ*λ* = 10 nm. The approximation error when fitting literature data for *α*^28,38^ with this basis is well below the measurement uncertainties.

The distributions *q*(*c*_Hb_) and *r*(*R*) are modelled by a normal distribution and log-normal distribution, respectively, each of which has two parameters *μ* and *σ*. Hence the parameter vector of the joint probability distribution is

7 $${\boldsymbol{\theta }}\,:\,={({\mu }_{{c}_{{\rm{Hb}}}},{\sigma }_{{c}_{{\rm{Hb}}}},{\mu }_{R},{\sigma }_{R})}^{T}.$$

The function which implements Eq. (5), using numerical integration of *c*_Hb_ and *R*, the Mie solution and Eq. (6) is referred to as $\bar{{\mathscr{C}}}(\lambda ;{\boldsymbol{a}},{\boldsymbol{\theta }})$. To account for errors of the number concentration of cells from volumetric dilution and measurements of the hematology analyser, a prefactor is included and the mathematical forward model for the spectral extinction reads

8 $${\mathscr{M}}(\lambda ;{\boldsymbol{\psi }})\,:\,=(1+\eta )\,\bar{{\mathscr{C}}}(\lambda ;{\boldsymbol{a}},{\boldsymbol{\theta }}),$$

with parameter vector

9 $${\boldsymbol{\psi }}\,:\,={(\begin{array}{c}{{\boldsymbol{a}}}^{T},{\theta }_{2},{\theta }_{3},{\theta }_{4},\eta \end{array})}^{T}.$$

Note that the parameter ${\theta }_{1}={\mu }_{{c}_{{\rm{Hb}}}}={\rm{MCHC}}$ is not a free parameter, but fixed to the value obtained from the CBC. This was done because the MCHC and the absolute value of the real RI increment *α*(*λ*) have a very similar effect on the model for the spectral extinction measurements leading to ambiguities and inconsistencies of the regression results of the RI increment. Using the MCHC as a free parameter in the least-squares optimisation described below worked well for the majority of datasets. However, for a few of the samples hematological parameters in disagreement with the CBC were found when this approach was applied to analyse measurements. Hence the MCHC was held constant in the analysis of all datasets. This yields consistent regression results for all data sets and made the results also substantially more robust against perturbations in the measurements.

A secondary model ${ {\mathcal M} }^{{\rm{CBC}}}({\boldsymbol{\theta }})$ computes the vector of blood count parameters ***z*** = (MCV, RDW)^*T*^ from ***θ***. Data analysis consists in minimising the cost function

10 $${\chi }^{2}({\boldsymbol{\psi }})\,:\,=\sum _{i=1}^{N}{w}_{i}\,{[ {\mathcal M} ({\lambda }_{i};{\boldsymbol{\psi }})-{y}_{i}]}^{2}+\sum _{j=1}^{2}{w}_{j}^{{\rm{CBC}}}{[{ {\mathcal M} }_{j}^{{\rm{CBC}}}({\boldsymbol{\theta }})-{z}_{j}]}^{2},$$

where *y*_*i*_ are the measured extinction cross sections and *z*_*j*_ are the CBC measurements. Weights are set to *w*_*i*_ = 1/*u*(*y*_*i*_)^2^ and ${w}_{j}^{{\rm{CBC}}}=1/u{({z}_{j})}^{2}$, where the standard uncertainties of the extinction spectra *y*_*i*_ are estimated from repeated measurements. To find an optimal parameter vector ***ψ*** that minimises *χ*^2^ we applied the nonlinear least squares optimisation lsqnonlin in Matlab (Matlab R2018a, The MathWorks Inc.) using the trust-region algorithm.

Because the objective function *χ*^2^ may have several local minima, initial values of the parameter vector were sampled randomly around a given mean and the local optimisation was repeated several times. The coefficient vector ***a*** was initialised randomly such that the wide range of literature values reported for the real RI increment *α* was covered. Several percent of random variation were allowed for parameters of size and concentration distributions. For each dataset 25 to 50 random initial conditions were sampled and optimised. The parameter vector with the lowest *χ*^2^ was used as the result $\hat{{\boldsymbol{\psi }}}$.

More specifically, the coefficient vector ***a*** of the real RI increment *α*(*λ*) was initialised in a two-step process: (i) *α*(*λ*) was set to a constant ${\rm{c}}{\rm{o}}{\rm{n}}{\rm{s}}{\rm{t}}\in {\mathscr{N}}(0.235\,{\rm{m}}{\rm{L}}\,{{\rm{g}}}^{-1},0.04\,{\rm{m}}{\rm{L}}\,{{\rm{g}}}^{-1})$ and (ii) additional normally distributed independent random numbers were added to the *a*_*j*_, resulting in random dispersion features of 0.004 mLg^−1^ standard deviation for *α*(*λ*). ${\mathscr{N}}(\mu ,\sigma )$ describes normally distributed random numbers of mean *μ* and standard deviation *σ*. For the size and concentration distribution, the parameters ***θ*** were randomly initialised around those values obtained from the CBC with the MCHC fixed to the value of the CBC. Standard deviations of the Gaussian random numbers were set to 120 nm for mean(*R*) and 30 nm for std(*R*). The width of the Hb concentration distribution and the particle concentration error were sampled from ${\rm{s}}{\rm{t}}{\rm{d}}({c}_{{\rm{H}}{\rm{b}}})\in {\mathscr{N}}(7{\rm{ \% }}{\rm{M}}{\rm{C}}{\rm{H}}{\rm{C}},10\,{\rm{g}}\,{{\rm{L}}}^{-1})$ and $\eta \in {\mathscr{N}}(0,3{\rm{ \% }})$, respectively.

25 to 50 random initial conditions were sampled and the optimisation was run for 15 iterations. Afterwards the six parameter vectors with the lowest *χ*^2^ were further optimised for up to 150 iterations or until a given tolerance was met. The parameter vector with the lowest *χ*^2^ was used as the result $\hat{{\boldsymbol{\psi }}}$. Typically several initial conditions ended up in the same minimum, but other less deep local minima were found as well.

#### Uncertainty propagation

Using the Jacobi matrix J of the extinction cross section with respect to the parameters with entries

11 $${J}_{ij}=\frac{\partial  {\mathcal M} ({\lambda }_{i},{\boldsymbol{\psi }})}{\partial {\psi }_{j}}$$

and the Jacobi matrix of the CBC model

12 $${J}_{ij}^{{\rm{CBC}}}=\frac{\partial { {\mathcal M} }_{i}^{{\rm{CBC}}}({\boldsymbol{\psi }})}{\partial {\psi }_{j}},$$

the covariance matrix corresponding to the uncertainty of the optimal parameter vector $\hat{{\boldsymbol{\psi }}}$ is determined as

13 $${\rm{\Sigma }}(\hat{{\boldsymbol{\psi }}})={[{{\rm{J}}}^{T}{\rm{WJ}}+{{\rm{J}}}^{{{\rm{CBC}}}^{T}}{{\rm{W}}}^{{\rm{CBC}}}{{\rm{J}}}^{{\rm{CBC}}}]}^{-1}.$$

This accounts for the effects of noise in the measured spectra (contained in W) and the uncertainty of the hematological parameters from the CBC (contained in W^CBC^). It does not account for other sources of error such as model errors. The first *M*×*M* components of this (*M* + 4) × (*M* + 4) matrix correspond to the covariance matrix ${\rm{\Sigma }}(\hat{{\boldsymbol{a}}})$ of the expansion coefficients of *α*(*λ*). The expansion coeffocients ${\hat{{\boldsymbol{a}}}}^{({\rm{A}})},\ldots ,{\hat{{\boldsymbol{a}}}}^{({\rm{F}})}$ of the results of the measurements from volunteers A–F are available as Supplementary Data S2. The estimated covariance matrices are available as Supplementary Data S3, S4, S5, S6, S7 and S8. According to Eq. (6) the *N*-vector of real RI increments *α*_*i*_ = *α*(*λ*_*i*_) at all wavelengths is given by ${\boldsymbol{\alpha }}={\rm{G}}\,{\boldsymbol{a}}$, where G is the *N* × *M* matrix of all basis functions (available as Supplementary Data S9). Hence for volunteer *j* = A, …, F, the corresponding *N* × *N* covariance matrix ${\rm{\Sigma }}({\hat{{\boldsymbol{\alpha }}}}^{(j)})$ of the real RI increments at all *N* wavelengths follows accordingly, by multiplying the *M* × *M* matrix ${\rm{\Sigma }}({\hat{{\boldsymbol{a}}}}^{(j)})$ from left and right with G and its transpose, respectively. The corresponding standard deviations (i. e., square-roots of the diagonal elements) are shown in Fig. 2 as shaded bands. As can be seen, they do not quite account for the variation between the samples from volunteers A–F, indicating that this uncertainty estimate is incomplete and that other sources of error than spectral noise and CBC measurement uncertainties are more significant. To combine the results of the *P* = 6 measurements we took the average of the optimisation results ${\hat{{\boldsymbol{\alpha }}}}^{(A)},\ldots ,{\hat{{\boldsymbol{\alpha }}}}^{(F)}$ (available as Supplementary Data S1), i. e.,

14 $$\langle \hat{{\boldsymbol{\alpha }}}\rangle =\frac{1}{P}\sum _{j=A}^{F}{\hat{{\boldsymbol{\alpha }}}}^{(j)}.$$

The variances corresponding to the uncertainties of the result were estimated from the sample variance, i. e.,

15 $${\rm{\Sigma }}{(\langle \hat{{\boldsymbol{\alpha }}}\rangle )}_{ii}=\frac{1}{P(P-1)}\,\sum _{j={\rm{A}}}^{{\rm{F}}}\,{({\hat{\alpha }}_{i}^{(j)}-\langle {\hat{\alpha }}_{i}\rangle )}^{2}.$$

To assess the influence of the RI of the sphering reagent and its uncertainty we performed an additional analysis assuming for the suspending fluid ${n}_{{\rm{m}}}(\lambda )={n}_{{{\rm{H}}}_{{\rm{2}}}{\rm{O}}}(\lambda )$ instead of ${n}_{{\rm{m}}}(\lambda )={n}_{{{\rm{H}}}_{{\rm{2}}}{\rm{O}}}(\lambda )+{\rm{\Delta }}n$ as done before with Δ*n* = 0.002. The resulting *α*(*λ*) is lower by 6 × 10^−3^ mLg^−1^ without any significant wavelength-dependence. Hence the sensitivity of the optimisation result to the numerical value of the RI of *n*_m_(*λ*) is

16 $$\frac{{\rm{\Delta }}\alpha }{{\rm{\Delta }}n}=\frac{6\times {10}^{-3}\,{\rm{mL}}\,{{\rm{g}}}^{-1}}{2\times {10}^{-3}}=3\,{\rm{mL}}\,{{\rm{g}}}^{-1}.$$

To account for the uncertainty of the RI of the sphering reagent of *u*[*n*_m_(*λ*)] = 3 × 10^−4^, stemming from the measurement of Δ*n* with an Abbe refractometer, an additional uncertainty term

17 $${u}_{{n}_{{\rm{m}}}}[\alpha (\lambda )]=\frac{{\rm{\Delta }}\alpha }{{\rm{\Delta }}n}\,u[{n}_{{\rm{m}}}(\lambda )]=9\times {10}^{-4}{\rm{mL}}\,{{\rm{g}}}^{-1}$$

is included. The total estimated standard uncertainty of the result is thus

18 $$u[\alpha ({\lambda }_{i})]=\sqrt{{u}_{{n}_{{\rm{m}}}}{[\alpha ({\lambda }_{i})]}^{2}+{\rm{\Sigma }}{(\langle \hat{{\boldsymbol{\alpha }}}\rangle )}_{ii}}.$$

## Supplementary information

Supplementary Dataset 1

Supplementary Dataset 2

Supplementary Dataset 3

Supplementary Dataset 4

Supplementary Dataset 5

Supplementary Dataset 6

Supplementary Dataset 7

Supplementary Dataset 8

Supplementary Dataset 9

## Data Availability

The datasets generated during the current study are available from the corresponding author on reasonable request. All data analysed during this study are included in this published article (and its Supplementary Information files).
